# Efficacy and safety of ultra-short wave diathermy on COVID-19 pneumonia: a pioneering study

**DOI:** 10.3389/fmed.2023.1149250

**Published:** 2023-06-05

**Authors:** Liangjiang Huang, Qian Li, Sayed Zulfiqar Ali Shah, Mohammad Nasb, Iftikhar Ali, Bin Chen, Lingfeng Xie, Hong Chen

**Affiliations:** ^1^Department of Rehabilitation Medicine, Tongji Hospital, Tongji Medical College, Huazhong University of Science and Technology, Wuhan, China; ^2^WHO Collaborating Center for Training and Research in Rehabilitation, Tongji Hospital, Tongji Medical College, Huazhong University of Science and Technology, Wuhan, China; ^3^Paraplegic Center, Hayatabad, Peshawar, Pakistan; ^4^Department of Radiology, Tongji Hospital, Tongji Medical College, Huazhong University of Science and Technology, Wuhan, China

**Keywords:** coronavirus disease 2019, ultra-short wave diathermy, rehabilitation, systemic inflammatory response scale, time to clinical recovery, pneumonia, pulmonary fibrosis

## Abstract

**Background:**

The ultra-short wave diathermy (USWD) is widely used to ameliorate inflammation of bacterial pneumonia, however, for COVID-19 pneumonia, USWD still needs to be verified. This study aimed to investigate the efficacy and safety of USWD in COVID-19 pneumonia patients.

**Methods:**

This was a single-center, evaluator-blinded, randomized controlled trial. Moderate and severe COVID-19 patients were recruited between 18 February and 20 April 2020. Participants were randomly allocated to receive USWD + standard medical treatment (USWD group) or standard medical treatment alone (control group). The negative conversion rate of SARS-CoV-2 and Systemic Inflammatory Response Scale (SIRS) on days 7, 14, 21, and 28 were assessed as primary outcomes. Secondary outcomes included time to clinical recovery, the 7-point ordinal scale, and adverse events.

**Results:**

Fifty patients were randomized (USWD, 25; control, 25), which included 22 males (44.0%) and 28 females (56.0%) with a mean (SD) age of 53 ± 10.69. The rates of SARS-CoV-2 negative conversion on day 7 (*p* = 0.066), day 14 (*p* = 0.239), day 21 (*p* = 0.269), and day 28 (*p* = 0.490) were insignificant. However, systemic inflammation by SIRS was ameliorated with significance on day 7 (*p* = 0.030), day 14 (*p* = 0.002), day 21 (*p* = 0.003), and day 28 (*p* = 0.011). Time to clinical recovery (USWD 36.84 ± 9.93 vs. control 43.56 ± 12.15, *p* = 0.037) was significantly shortened with a between-group difference of 6.72 ± 3.14 days. 7-point ordinal scale on days 21 and 28 showed significance (*p* = 0.002, 0.003), whereas the difference on days 7 and 14 was insignificant (*p* = 0.524, 0.108). In addition, artificial intelligence-assisted CT analysis showed a greater decrease in the infection volume in the USWD group, without significant between-group differences. No treatment-associated adverse events or worsening of pulmonary fibrosis were observed in either group.

**Conclusion:**

Among patients with moderate and severe COVID-19 pneumonia, USWD added to standard medical treatment could ameliorate systemic inflammation and shorten the duration of hospitalization without causing any adverse effects.

**Clinical Trial Registration**: chictr.org.cn, identifier ChiCTR2000029972.

## Introduction

The outbreak of the coronavirus disease 2019 (COVID-19) pandemic has prompted efforts to manage the threat to the well-being of populations worldwide (1–4). The new variants of SARS-CoV2 have emerged and have spread widely worldwide (5, 6). In response to the critical demand for high-quality clinical guidance at the peak of the outbreak in China, guidelines were published to clarify that physical therapy could play an important role in managing COVID-19 (7–10).

Ultra–short wave diathermy (USWD) and short-wave diathermy (SWD) are both forms of radiofrequency radiation energy with high-frequency electrotherapy (27.12or 40.68 MHz) as the commonly used tools of physical therapy and rehabilitation (11). The USWD and SWD have been used for decades in the field of rehabilitation for managing a variety of conditions: such as spontaneous pneumothorax (12, 13), knee osteoarthritis (14, 15), pelvic inflammation (16), peptic ulcer (17), peripheral myelinopathies (18), lung injury (19), and respiratory infectious diseases, etc. (20–22). USWD has similar therapeutic properties to SWD but the former got deeper penetration, less heat production, and is considered more suitable for the acute phase (23). The therapeutic effects of USWD and SWD on the body parts include producing deep heat (about 5 cm under the skin), inducing vasodilation, enhancing cellular activity, attenuating inflammation, and reducing pain (18, 24–29). It has previously been proven that raising the temperature decreases the activity and viability of the viruses (30). Thus, based on earlier studies, the utilization of short-wave diathermy could aid in such infectious conditions. During the outbreak of severe acute respiratory syndrome (SARS) in 2003, USWD was widely used by rehabilitation professionals in China to reduce pulmonary inflammation, and Zhang et al. (31) evaluated the efficacy of USWD and conventional therapy, finding that USWD could accelerate recovery and reduce the length of hospital stay in 38 patients with SARS. USWD has also been proven helpful for acute lung injury in rats by attenuating inflammation through the modulation of macrophage polarization (18). However, its application for COVID-19 pneumonia still needs to be validated.

In order to find robust evidence for the efficacy and safety of USWD in COVID-19 patients, we designed a randomized controlled trial to investigate the application of USWD in managing COVID-19 pneumonia.

## Methods and materials

### Trial design and ethical considerations

**Figure 1:**
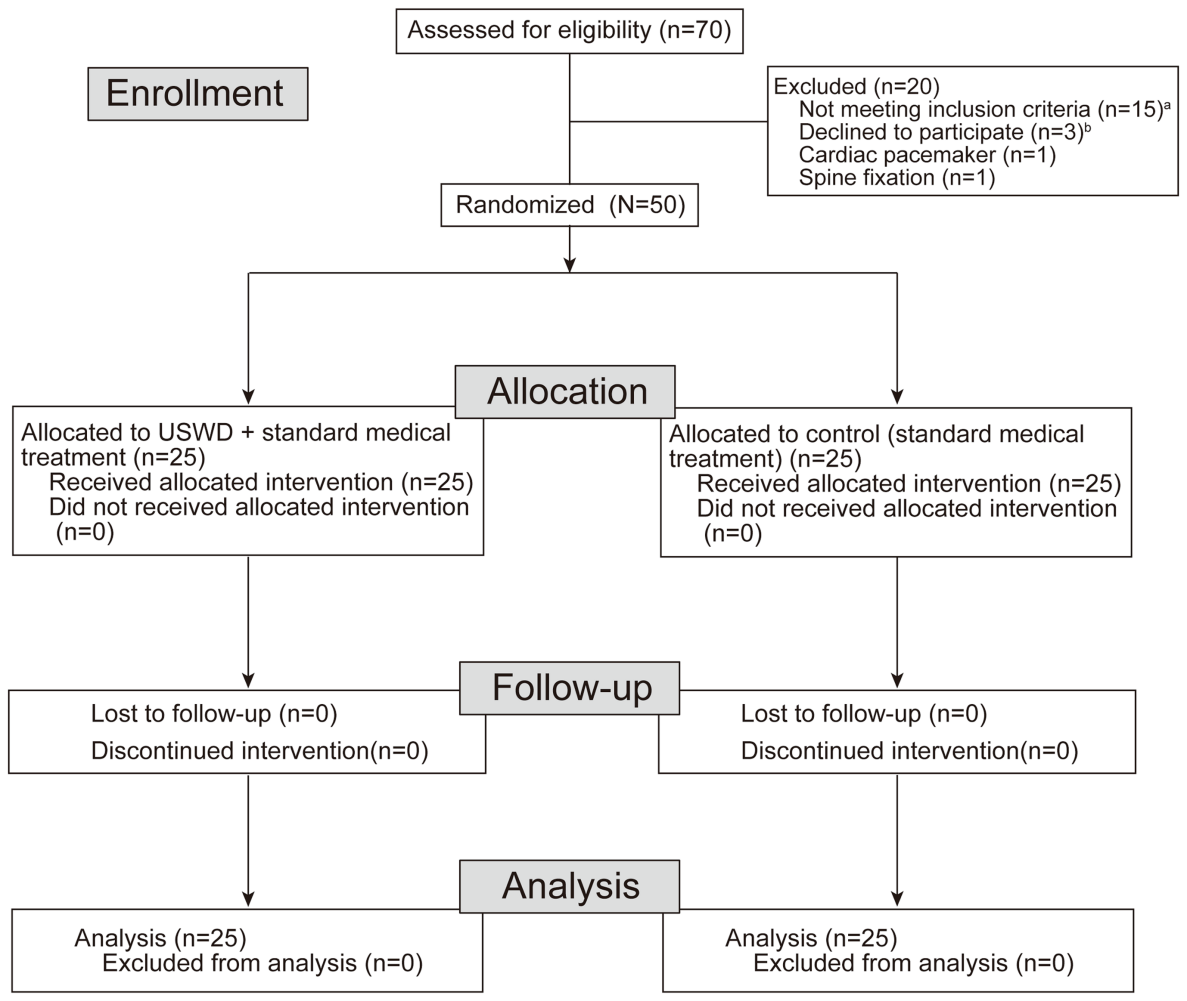
Flow chat of participant screening and randomization. ^a^Amang 15 exculded, 5 tested negatives for SARS-CoV-2, 3 were positive for other pathogens, 7 needed ICU care. ^b^Three patients declined to participate during precliminary screening because of personal reasons.

This single-center, evaluator-blinded, two-arm (1:1 ratio) parallel design, superiority randomized controlled trial was approved by the ethics committee of the Tongji Hospital, Tongji Medical College, Huazhong University of Science and Technology, Wuhan, China (certificate of approval number: TJ-C20200127), and prospectively registered on 17 February 2020, with the Chinese Clinical Trials Registry (Identifier: ChiCTR2000029972). The study was conducted in accordance with the relevant regulations and guidelines of good clinical practice and the Declaration of Helsinki. Patient recruitment, randomization, and study events are visually described in the CONSORT flow diagram (Figure 1). Participants were recruited between 18 February 2020 and 20 April 2020. Before randomization, written and verbal informed consent was obtained, and informative essays that clearly showed the risks and the supposed benefits accompanying the participation were provided to each patient.

### Participants

Patients of all genders admitted at the Tongji Hospital of Huazhong University of Science and Technology (Wuhan, China), and qualifying the following criteria and were recruited in this study as follows: (1) aged 18 to 65 years, (2) positive SARS-CoV-2 nucleic acid test by nasopharyngeal swabs, and (3) multiple patchy ground-glass shadows or other typical manifestations in both lungs diagnosed in lung computed tomography (CT). The exclusion criteria were: (1) positive tests for other pathogens, such as influenza, tuberculosis, mycoplasma, etc., (2) patients with metal implants or pacemakers, (3) requiring mechanical ventilation, (4) multiple organ failure requiring intensive care unit (ICU) monitoring and treatment, (5) bleeding tendency or active bleeding in the lungs, (6) shock, (7) cancer and severe underlying diseases, (8) severe cognitive impairment, (9) pregnancy or lactation, (10) those without informed consent, and (11) those with other contraindications to ultra–short wave diathermy. Subjects who met any of the exclusion criteria were not enrolled in this study.

All participants were classified as moderate or severe COVID-19 according to the severity of the disease (Classification according to the sixth edition of COVID-19 Diagnosis Guidelines released by China’s National Health Commission). The detailed classification criteria of moderate and severe cases were as, moderate: COVID-19 patients with fever and respiratory symptoms (such as cough, dyspnea, etc.) with CT findings of pneumonia, severe: COVID-19 patients meeting any of the following three signs, (1) respiratory rate (RR) ≥ 30 times/min, (2) oxygen saturation (SpO_2_) ≤93% at rest, (3) Arterial oxygen tension/fractional inspired oxygen ratio (PaO_2_/FiO_2_) ≤ 300 mmHg (1 mmHg = 0.133 kPa).

### Randomization, allocation, and blinding

A statistician, who was not a part of the study, created an online randomization plan on www.randomization.com using the permuted blocks method with small blocks of various sizes. A total of 50 patients were randomized to either an experimental USWD group (n = 25) or a control group (*n* = 25). This was an assessor-blinded, controlled study, and because of the nature of the interventions, it could not be a therapist-or patient-blinded study; however, a well-trained healthcare team comprising two evaluators, two statisticians, and two data collectors were blinded to the groups/treatment allocation. The outcomes were independently documented based on a mutual consensus between the data collectors (Figure 1).

### Sample size

*A priori* sample size calculation was performed using GPower software version 3.1 (Düsseldorf, Nordrhein-Westfalen, Germany) based on the mean values of the length of clinical recovery from a previous SARS study (31), we estimated that with 80% power, 5% two-sided type I error rate, and an effect size of 0.72, enrolment of 62 participants should be sufficient to detect a statistically significant between-group difference of 6.6 days in the length of recovery from symptoms. However, four more participants were included in the total sample size to manage the expected 5% dropouts, making the total sample size 66 (33 participants in each group). We could not find a study with a similar intervention, reporting the primary variables as our study to calculate the required sample size more accurately.

### Intervention

The control group received the standard medical treatment as recommended by the sixth edition of the Chinese COVID-19 Diagnosis Guidelines, which included medical care, oxygen therapy, fluid suppletion, nonsteroidal anti-inflammatory drugs (NSAIDs) with analgesic, anti-inflammatory, and antipyretic properties, antiviral drugs, and sufficient antibiotics when combined with bacterial infection. The experimental group (USWD) received the nationally recommended standard medical treatment in addition to the USWD. The USWD was performed through the application of ultra-short wave therapy electrodes on the anterior and posterior parts of the trunk for 10 min, twice daily for 12 consecutive days. The ultra-short wave therapy machine specifications and details are as follows: ultra-short wave electricizer (Dajia DL-C-C, factory no: BE1003094, A.C. power 220 V, 50 Hz, 700VA, Shantou Medical Equipment Factory Co., Ltd., China, Guangdong). We applied USWD in continuous mode with a frequency of 27.12 MHz and a power of 200 W. With these parameters, the patient would feel mild or no heat. In contrast, the control group received only the nationally recommended standard treatment. Moreover, the testing of USWD machine output, disinfection of the machine and electrodes, wearing masks, and protective suits, and testing of the patient’s skin sensation before the intervention were performed to ensure treatment safety.

### Data collection tools

The data collection forms developed for this trial consisted of medical history forms to obtain relevant medical history, case report form (CRF) to collect treatment-related data, and adverse events form to collect data on the occurrence of any adverse event during the trial.

### Clinical observation

The clinical assessment was performed at five-time points: at baseline, and on days 7, 14, 21, and 28 of treatment. The evaluation details are as follows:

Before treatment: (I) Evaluation and recording of demographic data, vital signs (pulse, respiration, blood pressure, body temperature), blood oxygen saturation, and vital capacity, (II) Medical history: including current medical history, past medical history, and drug-allergy history, (III) Laboratory tests: SARS-CoV-2 nucleic acid test by pharyngeal swabs RT-PCR, Complete blood count (CBC), Lactate dehydrogenase (LDH), (IV) Radiological examination: Chest CT, (V) Other tests: ECG, (VI) Combined medications, and (VII) Symptoms evaluation: Completing the 7-category ordinal scale and SIRS scores.
Treatment and follow-up period (days 7, 14, 21, and 28): (I) Evaluation and recording of vital signs (pulse, respiration, blood pressure, body temperature), blood oxygen saturation, and vital capacity, (II) Laboratory blood tests: SARS-CoV-2 nucleic acid test by pharyngeal swabs RT-PCR, Complete blood count (CBC), Lactate dehydrogenase (LDH), serum enzyme levels of alanine aminotransferase (ALT) and aspartate aminotransferase (AST), and international normalized ratio (INR), (III) Radiological examination, in some patients chest CT scans were not performed frequently due to radiation hazards, but only underwent CT scans after treatment, mainly on day 14, (IV) Other tests and assessments. ECG, and (V) Symptoms evaluation, completion of SIRS scores (including heart and respiratory rate, mean arterial pressure, SpO_2_, body temperature, white blood cells, and level of consciousness), and the 7-category ordinal scale.

## Outcome measurements

### Primary outcomes

The primary outcomes were the negative conversion rate of SARS-CoV-2 nucleic acid test by reverse transcription PCR (RT-PCR) and Systemic Inflammatory Response Scale (SIRS) (Supplementary Appendix 1) in the USWD group on days 7, 14, 21, and 28 of treatment, compared with those in the control group (standard medical treatment alone).

### Secondary outcomes

The secondary outcomes included the clinical outcomes (the time to clinical recovery, 7-point ordinal scale), lung CT images, combined medications, and laboratory blood tests in the USWD group at days 7, 14, 21, and 28 after treatment, compared with those in the control group. An artificial intelligence (AI)-aided CT image analysis tool was applied for the quantitative analysis of the infected lung area proportion and volume. The quantification of lung pneumonia in COVID-19 patients was measured from chest CT by using an available deep learning approach described detailedly before (32). Quantitative analysis of lung opacification was performed using a commercial deep learning software in InferScholarTM Center (InfervisionTM, Beijing, China).

The definitions of clinical recovery were as follows: (1) temperature returned to normal for more than 3 days; (2) significant improvement in respiratory symptoms (such as cough and breathing difficulty); (3) significant decrease in acute exudative lesions on lung CT imaging; and (4) two consecutive negative nucleic acid test results with nasopharyngeal swabs (the sampling interval was at least 24 h).

### Safety

Adverse events, assessment of vital signs, abnormal serum laboratory tests and clinical complications during the intervention were collected in both groups.

### Statistical analysis

We planned to enroll 66 participants according to our protocol; however, due to the subsequent unavailability of COVID-19 patients at our hospital, we had to restrict the study to 50 patients. All statistical analyses were performed using Statistical Package for Social Sciences (SPSS) version 25.0 and GraphPad Prism 8. An intention-to-treat analysis was used. Data normality was assessed with the Kolmogorov–Smirnov, and Shapiro–Wilk tests. Continuous variables are presented as mean (standard deviation, SD) in case of normal distribution of data or median (inter-quartile range, IQR) in case of non-normal distribution, while categorical variables are presented as count (%). Descriptive statistics (mean, frequencies, and percentages) were calculated for demographic variables and primary and secondary variables in the study. Baseline and post-intervention comparisons between the USWD and control groups were performed using an independent samples *t*-test and Mann–Whitney statistics based on the normality results of the data. The proportions of categorical variables were compared using Fisher’s exact test/chi-square test. The Chi-square test was used for the evaluation of the 7-point ordinal scale, and the Mann–Whitney test was used for the SIRS scale (treated as ordinal scales). A difference-in-difference (D-in-D) analysis was used to analyze the AI-assisted CT scan data. Patients who failed to reach the negative conversion of SARS-CoV-2 by the cut-off date of the analysis were considered as right-censored at the last visit date. All patients were treated after the completion of follow-up (28 days).

## Results

### Patient demographics and clinical characteristics

A total of 70 patients were screened in this study, 20 were excluded for reasons and finally, 50 subjects were eligible to be enrolled, and randomized for this study. The CONSORT flow diagram is shown in Figure 1. Of the 50 enrolled participants, 22 (44.0%) were men and 28 (56.0%) were women, with a mean age (SD) of 53 ± 10.69 years. With 30 (60%) moderate and 20 (40%) severe cases, the USWD group contained more patients with severe conditions (52%) than the control group (28%), The median duration between onset and admission were 21 (13–27.0) days. The majority of the participants were non-smokers (86.0%), and 34.0% had co-morbid conditions (Table 1), such as diabetes (22%), hypertension (20%), and cardiovascular diseases (8%). Fever (90%), breathing difficulty (56%), dry cough (50%), diarrhea (34%), and fatigue (24%) were the top five most common symptoms reported on presentation (Table 2). Moreover, most of the patients had a dry cough (50%), while very few had a productive cough (14%). The baseline clinical characteristics of all participants are shown in Tables 1, 2, and Supplementary Table S1. Both groups were balanced at baseline with insignificant differences in demographic data, clinical features, disease severity, and laboratory tests.

**Table 1:** The demographics, severity, and baseline characteristics.

Items	Overall cohort (*n* = 50)	USWD group (*n* = 25)	Control group (*n* = 25)
Age (Mean ± SD)	53 ± 10.69	53 ± 9.29	54 ± 12.08
Gender (Male/Female)	22/28	11/14	11/14
Smoke, n/ N (%)	7/50 (14)	3/25 (12)	4/25 (16)
Severity of disease			
Moderate, *n*/N (%)	30/50 (60)	12/25 (48)	18/25 (72)
Severe, *n*/N (%)	20/50 (40)	13/25 (52)	7/25 (28)
Interval between onset and admission, median (IQR), d	21 (13–27.0)	18 (8–35.5)	13 (8–23.0)
Comorbidities, n/N (%)	17/50 (34)	8/25 (32)	9/25 (36)
SIRS score. Median (IQR)	2.5 (1–5.0)	3 (2–5)	2 (1–4.5)
7-point ordinal scale, No. (%)			
1. Not hospitalized with the resumption of normal activities	0 (0)	0 (0)	0 (0)
2. Not hospitalized, but unable to resume normal activities	0 (0)	0 (0)	0 (0)
3. Hospitalized, not requiring supplemental oxygen	4 (8)	3 (12)	1 (4)
4. Hospitalized, requiring supplemental oxygen	46 (92)	22 (88)	24 (96)
5. Hospitalized, requiring high-flow oxygen therapy or non-invasive mechanical ventilation	0 (0)	0 (0)	0 (0)
6. Hospitalized, requiring ECMO, invasive mechanical ventilation, or both	0 (0)	0 (0)	0 (0)
7. Death	0 (0)	0 (0)	0 (0)

**Table 2:** The comorbidities and symptoms at baseline.

Item	Overall cohort	USWD group	Control group
Coexisting diseases *n*/N (%)			
Cardiovascular disease	4/50 (8)	1/25 (4)	3/25 (12)
Hypertension	10/50 (20)	4/25 (16)	6/25 (24)
Diabetes	11/50 (22)	4/25 (16)	7/25 (28)
Stroke sequelae	1/50 (2)	0/25 (0)	1/25 (4)
Gout	2/50 (4)	1/25 (4)	1/25 (4)
arthritis	1/50 (2)	0/25 (0)	1/25 (4)
Signs and symptoms *n*/N (%)			
Fever	45/50 (90)	24/25 (96)	21/25 (84)
Chills	4/50 (8)	1/25 (4)	3/25 (12)
Muscle ache	12/50 (24)	4/25 (16)	8/25 (32)
Chest Pain	5/50 (10)	1/25 (4)	4/25 (16)
Breathing difficulty	28/50 (56)	13/25 (52)	15/25 (60)
Dry Cough	25/50 (50)	14/25 (56)	11/25 (44)
Productive Cough	7/50 (14)	2/25 (8)	5/25 (20)
Dyspnea	2/50 (4)	0/25 (0)	2/25 (8)
Fatigue	12/50 (24)	4/25 (16)	8/25 (32)
Headache	3/50 (6)	2/25 (8)	1/25 (4)
Palpitation	1/50 (2)	1/25 (4)	0/25 (0)
Diarrhea	17/50 (34)	7/50 (14)	10/25 (40)
Abdominal pain	1/50 (2)	0/25 (0)	1/25 (4)
Vomiting	4/50 (8)	0/25 (0)	4/25 (16)

### The negative conversion rate of SARS-CoV-2 nucleic acid

**Figure 2:**
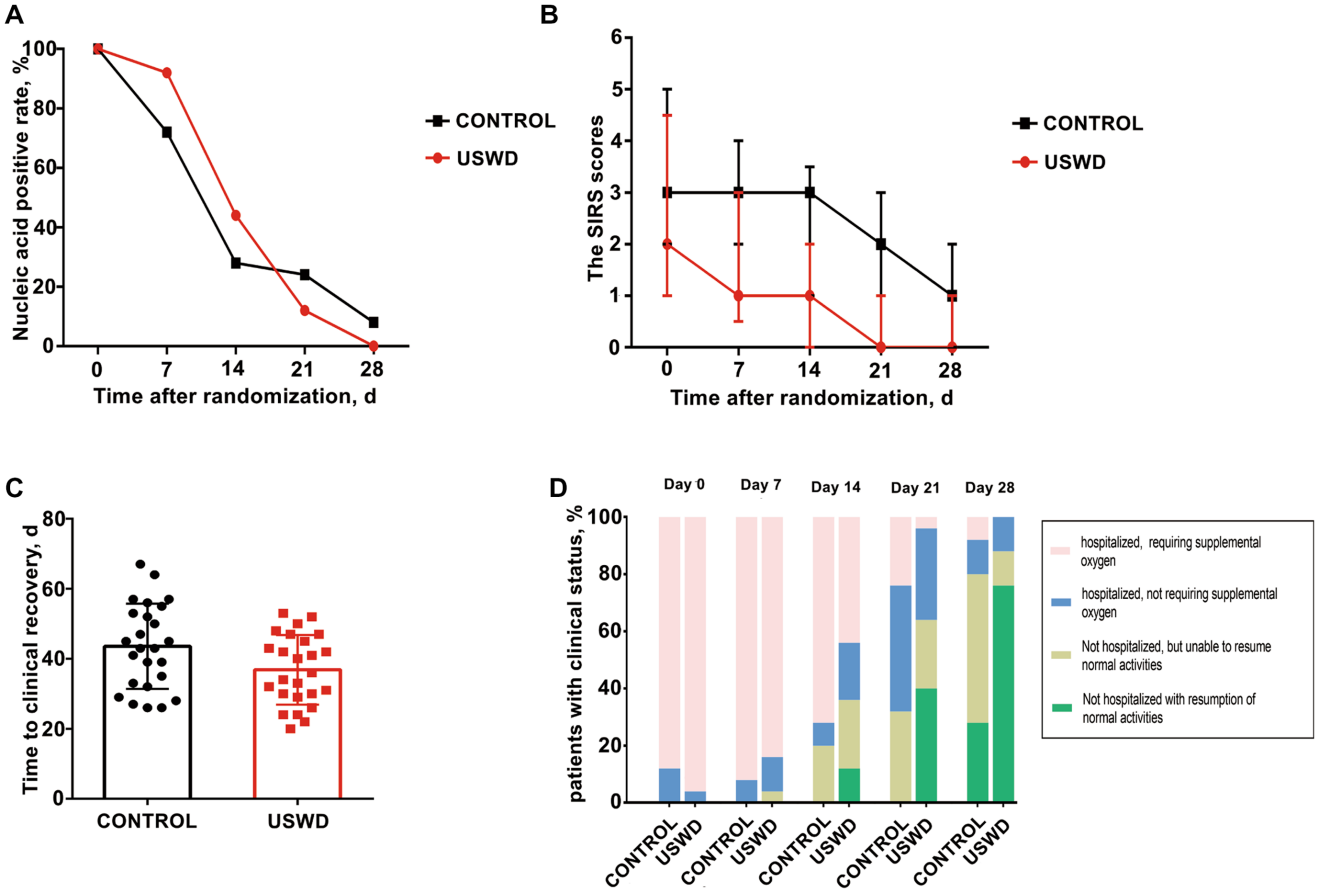
Primary and secondary outcomes at baseline, on days 7, 14, 21 and 28 by treatment group. **(A)** The SARS-CoV-2 nucleic acid negative conversion rate showed no significant difference between the USWD and control group at day 7 (*p* = 0.239), day 14 (*p* = 0.269), and day 28 (*p =* 0.490). **(B)** The clinical condition on SIRS score showed statistically significant difference on day 7 (*p* = 0.030), day 14 (*p* = 0.002), day 21 (*p* = 0.003) and day 28 (*p* = 0.011). **(C)** Time to clinical recovery in the USWD group was significantly shortened comparing with the control group (*p* = 0.037). **(D)** Clinical status on 7-point ordinal scale on study days 21 and 28 showed significance (*p* = 0.002, 0.003), whereas the difference at day 7 and 14 was insignificant (*p* = 0.524, 0.108).

In this study, we continuously conducted nucleic acid tests at least once weekly. The SARS-CoV-2 nucleic acid test negative conversion rate showed no significant difference between the USWD and control groups at days 7 (*p* = 0.066), 14 (*p* = 0.239), 21 (*p* = 0.269), and 28 (*p* = 0.490) (Table 3; Figure 2A).

**Table 3:** Primary and secondary clinical outcomes.

Variables	USWD group (*n* = 25)	Control group (*n* = 25)	*p*-value
Primary clinical outcomes			
Viral nucleic acid negative rate, No./total (%)			
At day 7	2/25 (8)	7/25 (28)	0.066
At day 14	14/25 (56)	18/25 (72)	0.239
At day 21	22/25 (88)	19/25 (76)	0.269
At day 28	25/25 (100)	23/25 (92)	0.490
SIRS scale (IQR)			
At day 7	1 (0.5–3)	3 (2–4)	0.030
At day 14	1 (0–2)	3 (1–3.5)	0.002
At day 21	0 (0–1)	2 (1–3)	0.003
At day 28	0 (0–1)	1 (0–2)	0.011
Secondary outcomes			
Time to clinical recovery (Mean ± SD), d	36.84 ± 9.93	43.56 ± 12.15	0.037
7-point scale on day 28, No./total (%)			0.003
1. Not hospitalized with the resumption of normal activities	19/25 (76)	7/25 (28)	
2. Not hospitalized, but unable to resume normal activities	3/25 (12)	13/25 (52)	
3. Hospitalized, not requiring supplemental oxygen	3/25 (12)	3/25 (12)	
4. Hospitalized, requiring supplemental oxygen	0/25 (0)	2/25 (8)	
5. Hospitalized, requiring high-flow oxygen therapy or non-invasive mechanical ventilation	0/25 (0)	0/25 (0)	
6. Hospitalized, requiring ECMO or invasive mechanical ventilation	0/25 (0)	0/25 (0)	
7. Death	0/25 (0)	0/25 (0)	
Patients of antiviral drugs used, *n*/N (%)	23/25 (92)	22/25 (88)	0.637

Ultra shortwave diathermy (USWD), Systemic Inflammatory Response Scale (SIRS),

### Clinical status of patients

Antiviral treatments were widely used in our study, there were 22 (88%) in the control group and 23 (92%) in the USWD group receiving different types of antiviral drugs, mainly oseltamivir, and abidol. The SIRS scores, which reflect patients’ present clinical condition, were statistically significantly different between the two groups at days 7 (*p* = 0.030), 14 (*p* = 0.002), 21 (*p* = 0.003), and 28 (*p* = 0.011) (Table 3; Figure 2B). The time to clinical recovery (days) in the USWD group was (6.72 ± 3.14) days shorter than that in the control group (36.84 ± 9.93 vs. 43.56 ± 12.15, *p* = 0.037). Moreover, the 7-point ordinal scale after intervention on days 21, and 28 also showed significant differences between the two groups (*p* = 0.002, and *p* = 0.003, respectively). However, the difference on days 7 and 14 was not statistically significant (*p* = 0.524, *p* = 0.108) (Table 3; Figures 2C,D). These findings suggest the therapeutic efficacy of implementing USWD in patients with COVID-19 pneumonia.

### CT scans and quantitative analysis

**Figure 3:**
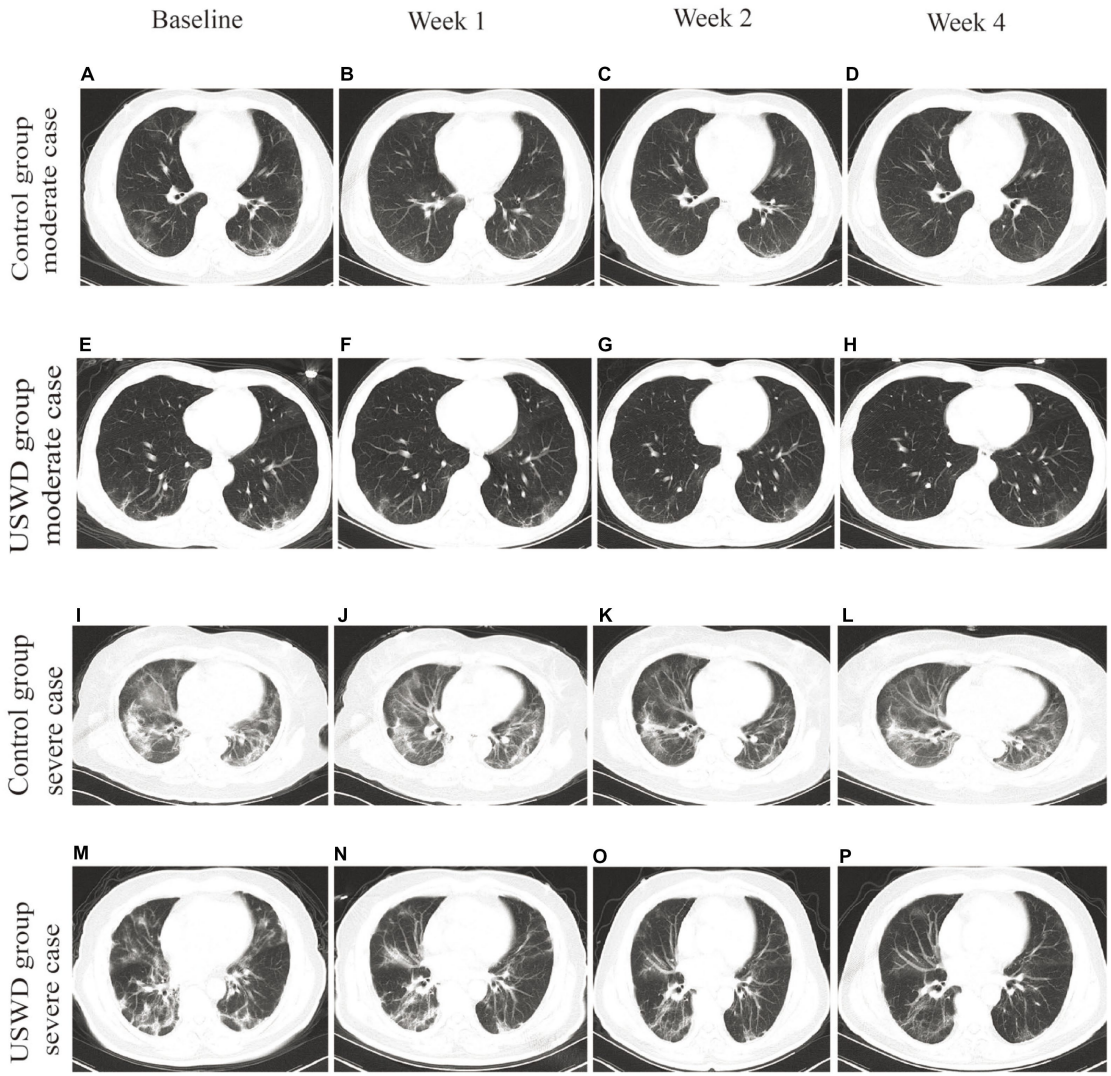
Chest CT images of moderate and severe cases in control and USWD group. **(A–D)** the CT scan of moderate cases in the control group show. **(E–H)** the CT scan of moderate cases in the USWD group. **(I–L)**: the CT scan of severe cases in the control group **(M–P)** the CT scan of severe cases in the USWD group.

In Figure 3, the CT images depicted the recovery progress in moderate and severe cases in both groups. Obvious multiple ground-glass opacities (GGOs) were observed, especially in the bilateral lower lung, with local thickening and adhesion of bilateral pleura. Pulmonary fibrosis, d stripe shadows, and consolidations could be seen in severe COVID-19 cases. Most of all, the worsening of pulmonary fibrosis was not observed in any group. The pulmonary fibrosis found before treatment was recovered in most of the patients (recovery: USWD = 14/15 and control = 16/18, *p* = 1.000).

The further artificial intelligence (AI)-aided quantitative analysis of CT images found that the mean volume of infected lung could reach 337.81 cm^3^ before treatment, while the lower lung had the worst infection areas and proportion (221.56 cm^3^, 65.6%) (Supplementary Table S2). Both groups showed improvements in the AI-aided CT imaging analysis. Following comparisons of quantitative values demonstrated USWD group got more decreased whole lung infection volume (69.7 cm^3^ vs. 46.2 cm^3^) and proportion (3.8% vs. 1.4%) than the control group without between-group significant differences (Table 4).

**Table 4:** Comparison of mean AI-assisted CT quantitative analysis of CT images between USWD and control group.

Variables	Control group			USWD group			D-in-D	P
	Before	After	Change^a^	Before	After	Change^a^		
Whole lung infection (%)	8.2 (8.1)	6.8 (8.1)	−1.4	12.8 (10.8)	8.7 (7.5)	−3.8	−2.29	0.5243
Whole lung infection V (cm^3^)	297.1 (268.7)	250.9 (256.6)	−46.2	395.0 (274.5)	325.3 (312.0)	−69.7	−0.163	0.9988
L lung infection (%)	6.8 (7.5)	5.6 (7.0)	−1.2	10.5 (9.2)	6.5 (7.0)	−4	−2.38	0.4607
L lung infection V (cm^3^)	114.2 (118.9)	100.2 (111.1)	−14	154.7 (117.1)	119.6 (141.5)	−35.1	−12.45	0.7995
L lung upper lobe infection (%)	5.1 (6.6)	4.0 (6.4)	−1.1	6.6 (7.5)	3.5 (3.8)	−3.1	−1.73	0.5107
L lung upper lobe infection V (cm^3^)	49.4 (69.8)	37.9 (56.7)	−11.5	56.0 (52.9)	38.0 (45.0)	−18	−3.72	0.8748
L lung lower lobe infection (%)	10.1 (12.2)	8.8 (9.8)	−1.3	17.3 (15.8)	12.2 (15.7)	−5.1	−3.07	0.5839
L lung lower lobe infection V (cm^3^)	61.7 (59.4)	66.5 (60.8)	4.8	98.7 (78.7)	83.3 (104.6)	−15.4	−14.53	0.6399
R lung infection (%)	9.9 (10.0)	7.8 (9.4)	−2.1	14.9 (13.3)	10.5 (9.9)	−4.4	−1.61	0.7158
R lung infection V (cm^3^)	182.9 (162.9)	148.8 (155.1)	−34.1	236.5 (179.7)	205.3 (191.5)	−31.2	17.60	0.7946
R lung upper lobe infection (%)	8.5 (10.8)	6.3 (9.1)	−2.2	11.0 (14.9)	7.8 (11.1)	−3.2	−0.41	0.9332
R lung upper lobe infection V (cm^3^)	58.0 (66.7)	45.0 (60.7)	−13	68.6 (79.1)	57.2 (74.8)	−11.4	7.46	0.7945
R lung middle lobe infection (%)	4.4 (6.4)	4.2 (7.2)	−0.2	9.2 (11.4)	7.8 (12.9)	−1.4	−0.67	0.8691
R lung middle lobe infection V (cm^3^)	16.1 (17.6)	15.2 (20.6)	−0.9	26.9 (27.1)	19.6 (27.4)	−7.3	−4.42	0.6391
R lung lower lobe infection (%)	15.8 (17.5)	12.4 (16.0)	−3.4	24.1 (19.6)	17.7 (16.0)	−6.4	−2.00	0.7769
R lung lower lobe infection V (cm^3^)	110.6 (100.2)	88.3 (91.5)	−22.3	145.3 (94.8)	130.6 (111.5)	−14.7	16.93	0.6594

Table 3 depicts the between groups comparison of different CT scan parameters before and after treatment. a = Average change in means before and after treatment, D-in-D=Difference-in-difference, V = volume, R = right, L = left.

### Adverse events (AEs), and complications

No serious AEs, deaths, permanent disability, neoplasia, or empyrosis cases were registered during the trial. Routine serum laboratory tests showed that all parameters were in almost equal and normal ranges in both groups. However, the WBC counts were significantly lower in the USWD group than in the control group (5.51 ± 1.38 vs. 6.56 ± 1.97). In contrast, the median (IQR) monocyte count was significantly higher in USWD than in the control group (8.92 [2.20] vs. 7.10 [1.15]), but the difference was of uncertain clinical importance (Table 5). Out of 50 patients, 22 each in the USWD and control groups had complications, 16 (64%) and 15 (60%) patients in the control and USWD group, respectively, had complications of bacterial pneumonia infections in the course and were treated with antibiotic drugs. Other complications included abnormal liver function test (LFT; 52% vs. 48%, *p* = 0.777), electrolyte imbalance (32% vs. 44%, *p* = 0.382), hyperfibrinogenaemia (44% vs. 48%, *p* = 0.777), and mild anemia (32% vs. 52%, *p* = 0.152) (Table 5). All complications were unrelated to USWD treatment and were not statistically different between the two groups.

**Table 5:** The laboratory values and complications in USWD and control groups.

Variables	USWD group (*n* = 25)	Control group (*n* = 25)	*p*-value
Major laboratory values after treatment			
Red blood cells 10^9^/L	4.05 (0.80)	4.03 (0.62)	0.620
White blood cells 10^9^/L	5.51 ± 1.38	6.56 ± 1.97	0.035
Neutrophil count 10^9^/L	2.99 (1.49)	3.83 (1.59)	0.071
Neutrophil percent %	58.49 ± 7.24	58.55 ± 9.37	0.980
Lymphocyte count 10^9^/L	1.60 (0.63)	1.79 (0.97)	0.207
Lymphocyte percent %	29.98 ± 6.96	28.79 ± 8.42	0.589
Monocyte count 10^9^/L	8.92 (2.20)	7.10 (1.15)	0.002
ALT U/L	23.00 (13.0)	24.00 (16.0)	0.771
AST U/L	23.00 (7.0)	19.00 (10.0)	0.264I
International normalized ratio (INR)	1.03 (0.09)	1.00 (0.06)	0.022
Complications, n/N (%)			
Bacterial pneumonia	15/25 (60)	16/25 (64)	0.771
Abnormal LFTs	13/25 (52)	12/25 (48)	0.777
Elevated AST	7/25 (28)	7/25 (28)	1.000
Elevated ALT	12/25 (48)	12/25 (48)	1.000
Electrolyte imbalance	8/25 (32)	11/25 (44)	0.382
Hyperfibrinogenemia	11/25 (44)	12/25 (48)	0.777
Anemia	8/25 (32)	13/25 (52)	0.152
Hypoalbuminemia	11/25 (44)	6/25 (24)	0.136
Abnormal blood coagulation	2/25 (8)	2/25 (8)	1.000
Renal insufficiency	4/25 (16)	1/25 (4)	0.349
Myocardial damage	1/25 (4)	1/25 (4)	1.000
No complications	3/25 (12)	3/25 (12)	1.000

LFTs, liver function tests; AST, Aspartate Aminotransferase; ALT, Alanine Aminotransferase.

## Discussion

USWD could induce vasodilation, increase blood flow, reduce inflammation, and decrease pain in a continuous mode (15, 18), suggesting that USWD might be beneficial for COVID-19 pneumonia. However, high-quality evidence to recommend the application of USWD in improving COVID-19 pneumonia is still lacking. To the best of our knowledge, this is the first randomized clinical trial investigating the efficacy of USWD treatment in COVID-19.

In this randomized clinical trial, we systematically investigated the therapeutic efficacy and safety of USWD in patients with moderate or severe COVID-19 pneumonia. The administration of USWD improved the clinical condition of patients with COVID-19 pneumonia who were hospitalized and required supplemental oxygen therapy. However, the SARS-CoV-2 negative conversion rate was not significantly increased by USWD, suggesting that USWD exerts therapeutic function independent of the direct antiviral effect. Surprisingly, after a 12-day course of USWD administered twice daily, there was a significant improvement in the mean scores of SIRS, an indicator of clinical condition. At the same time, USWD could shorten the course of COVID-19 pneumonia by (6.72 ± 3.14) days. These findings of this study are consistent with previous studies in 2003 during the SARS. Zhang LF (31) used USWD in 38 SARS pneumonia patients, and found that the administration of USWD accelerated pneumonia recovery and shortened the length of hospital stay. Some other studies with bacterial pneumonia patients treated with USWD showed similar results in clinical recovery as the findings in our study. He YG (33) found USWD could reduce inflammation, and promote lung tissue repair in children with bronchopneumonia. Du QP (34) applied USWD therapy in infants with pneumonia and reported that additional USWD reduced the duration period of symptoms, shortened the treatment course, and reduced the use of antibiotics. Moreover, Zhu Q (35) reported that USWD combined with standard medications could impart better properties to pulmonary function and clinical recovery. Our study provided further evidence of the effectiveness of USWD in the role of inflammation control, which suggests that USWD might be a potential therapeutic means for COVID-19 pneumonia.

Treatment with USWD, however, increased the number of monocytes in our study, which are an important component of the body’s immune system, although within normal range, and reduced the number of WBCs, which is a biological marker of inflammation. These findings are consistent with those of previous studies of the physiological effects of short-wave therapy (24), supporting the immune response to accelerate recovery. Thus, the administration of USWD at an early stage in pneumonia may stimulate and boost the body’s natural defenses against microorganisms (36).

Lung CT images could provide supportive assistance in the early diagnosis and monitoring of lung lesions in patients with COVID-19 pneumonia. Previously, there were concerns like USWD induces fibroblastic activity, and that the enriched oxygen environment could hypothetically increase the risk of pulmonary complications, such as pulmonary fibrosis. There was a theoretical hypothesis that the synergistic activity of USWD and high oxygen environment in COVID-19 pneumonia patients could cause or aggravate pulmonary fibrosis (26, 37, 38). In fact, some pre-clinical studies found that USWD could increase the extensibility of collagenous tissue (15), protect damaged lung tissue, and reduce pulmonary interstitial fibrosis (39, 40). Previous clinical studies have shown that USWD as adjuvant therapy in children and adults with pneumonia was effective and did not aggravate pulmonary fibrosis (41–43). In our study, the pulmonary fibrosis observed in CT before treatment was recovered in most of the patients, and worsening of pulmonary fibrosis was not observed in any patient. Overall, the finding of pulmonary fibrosis recovery could completely overcome the safety concerns of fibrosis in USWD.

Additionally, Lung opacification percentages and volume of the whole lung and five lobes were automatically quantified by using a deep learning algorithm (32). Traditional visual evaluation of CT scans is subjective, and its validity mainly depends on the radiologist’ experience. Quantitative analysis of the CT imaging using the artificial intelligence tool, such as deep learning, could provide an automatic and objective estimation to identify the severity, monitor disease progression and help understand the course of COVID-19 pneumonia (44, 45). We applied AI-aided CT assessment tools to compare the therapeutic effect on lung opacification between the two groups in this study, which made our research more rigorous.

### Strengths and limitations

The strength of our study is the rigorous design of our randomized controlled trial. As the cases in our study were from the early period of the pandemic outbreak when the virus was extremely virulent and there was no vaccine available, which was conducive to fully demonstrating the effect of USWD. Finally, USWD therapy was easy to apply and had few contraindications, it could economize the medical costs and may help to reduce the consumption of antibiotics or antivirals once widely applied in the future.

The major limitation of the present study was the relatively small sample size, and the follow-up period of only 28 days, which maybe not be long enough for severe COVID-19 patients. Moreover, many novel SARS-CoV-2 variants like delta and omicron had emerged, and the effectiveness of USWD for the new variants was uncertain. Given that the function of USWD was dependent on non-specific anti-inflammatory properties, USWD might conceivably be effective for different SARS-CoV-2 variants.

## Conclusion

USWD could not accelerate the SARS-CoV-2 nucleic acid negative conversion rate. However, the administration of USWD could significantly improve the clinical status and effectively shorten the length of hospitalization in patients with moderate and severe COVID-19 pneumonia, without aggravating pulmonary fibrosis. Further studies are necessary to understand the definite curative effects of USWD in COVID-19 pneumonia and other different pathogens.

## Data availability statement

The original contributions presented in the study are included in the article/Supplementary material, further inquiries can be directed to the corresponding author.

## Ethics statement

The studies involving human participants were reviewed and approved by The ethics committee of the Tongji Hospital, Tongji Medical College, Huazhong University of Science and Technology, Wuhan, China (certificate of approval number: TJ-C20200127). The patients/participants provided their written informed consent to participate in this study.

## Author contributions

HC contributed to the supervision, drafting, and finalizing of the study. LH, QL, and SS equally contributed to compiling and describing the results. Moreover, authors LH and QL contributed to designing the CRF, medical history forms development, data collection, and interpretation. SS and MN contributed to writing and formatting the manuscript. LX designed the USWD treatment protocol, while BC did the CT scan analysis. IA contributed to data analysis. All authors contributed to the article and approved the submitted version.

## Funding

The study was funded by the Key Research and Development program of Hubei province (No. 2022BCA028) and the Health Commission of Hubei Province (No. WJ2023M003).

## Conflict of interest

The authors declare that the research was conducted in the absence of any commercial or financial relationships that could be construed as a potential conflict of interest.

## Publisher’s note

All claims expressed in this article are solely those of the authors and do not necessarily represent those of their affiliated organizations, or those of the publisher, the editors and the reviewers. Any product that may be evaluated in this article, or claim that may be made by its manufacturer, is not guaranteed or endorsed by the publisher.
