# Supplementary material for: Efficacy and safety of ultra-short wave diathermy on COVID-19 pneumonia: a pioneering study
Source: Front Med (Lausanne). 2023 Jun 5;10:1149250. doi: 10.3389/fmed.2023.1149250 (PMC10277738; doi:10.3389/fmed.2023.1149250)
Supplement: Supplementary file 1 [file Data_Sheet_1.docx]

**Supplementary Table S1. Laboratory tests values before treatment**

| **Variables** | **Overall cohort** | **Control group** | **USWD group** |
| --- | --- | --- | --- |
| Red blood cells, 10^9^/L | 4.15(0.76) | 4.04(0.73) | 4.20(0.70) |
| White blood cells, 10^9^/L | 6.30(2.64) | 6.68(3.88) | 5.56(2.76) |
| Neutrophil count, 10^9^/L | 4.30(2.59) | 4.29(3.33) | 3.29(2.13) |
| Neutrophil percent, % | 64.55±13.66 | 67.52±13.41 | 61.57±13.51 |
| Lymphocyte count, 10^9^/L | 1.43±0.60 | 1.43±0.56 | 1.43±0.56 |
| Lymphocyte percent, % | 25.85(11.92) | 23.68(11.89) | 28.02(11.78) |
| Monocyte count, 10^9^/L | 8.23±2.95 | 7.77±2.79 | 8.68±3.09 |
| ALT, U/L | 32.67(22.71) | 29.00(16) | 26.00(13) |
| AST, U/L | 32.22(21.40) | 23.00(14.50) | 28.00(15.50) |
| INR | 2.86(12.86) | 1.05(0.10) | 1.02(0.12) |

Abbreviations: Systemic Inflammatory Response Scale (SIRS), Aspartate Aminotransferase (AST), Alanine Aminotransferase (ALT) International normalised ratio (INR)

**Supplementary Table S2. AI-assisted CT images analysis results of the total patients before and after treatment**

| **Parameter** | **Before treatment**  **(Mean± SD)** | **After treatment**  **(Mean± SD)** |
| --- | --- | --- |
| Whole lung infection proportion (%) | 10.19± 9.72 | 7.68± 7.56 |
| Whole lung infection volume (*cm^3^) | 337.81± 274.73 | 287.11±278.46 |
| Left lung infection proportion (%) | 8.46±8.50 | 6.01±6.78 |
| Left lung infection volume (*cm^3^) | 131.21±118.86 | 109.23± 122.82 |
| Left lung upper lobe infection proportion (%) | 6.40±7.13 | 4.27± 5.20 |
| Left lung upper lobe infection volume (*cm^3^) | 57.71± 61.91 | 43.39± 50.87 |
| Left lung lower lobe infection proportion (%) | 14.55±14.43 | 12.03± 12.86 |
| Left lung lower lobe infection volume (*cm^3^) | 85.28± 70.38 | 86.08± 83.12 |
| Right lung infection proportion (%) | 12.08± 11.87 | 9.06± 9.38 |
| Right lung infection volume (*cm^3^) | 204.78± 172.40 | 174.65± 170.88 |
| Right lung upper lobe infection proportion (%) | 11.52± 13.31 | 8.16± 10.16 |
| Right lung upper lobe infection volume (*cm^3^) | 74.67±73.22 | 58.88± 67.84 |
| Right lung middle lobe infection proportion (%) | 7.74±9.67 | 6.93± 10.78 |
| Right lung middle lobe infection volume (*cm^3^) | 24.12± 23.32 | 20.01±24.32 |
| Right lung lower lobe infection proportion (%) | 21.24± 18.68 | 16.63± 15.66 |
| Right lung lower lobe infection volume (*cm^3^) | 136.28± 95.35 | 120.61± 99.53 |

Supplementary Table 2. shows the AI-assisted CT scan parameters of the total population before and after treatment. The proportions and volumes of lung injury are shown. The data are shown as means and SD.
