# Supplementary material for: Efficacy and safety of ultra-short wave diathermy on COVID-19 pneumonia: a pioneering study
Source: Front Med (Lausanne). 2023 Jun 5;10:1149250. doi: 10.3389/fmed.2023.1149250 (PMC10277738; doi:10.3389/fmed.2023.1149250)
Supplement: Supplementary file 2 [file Data_Sheet_2.docx]

**Supplementary appendix**

**Systemic Inflammatory Response scale (SIRS)** is used for evaluation of clinical improvement based on heart rate, mean arterial pressure (MAP mmHg), respiratory rate/min, blood oxygen saturation (SpO2 %), body temperature（℃）, white blood cells (WBC *109/L), blood glucose (mmol/L) and level of consciousness (Aware/awake, Lethargy or irritability, shallow coma, coma, brain death). All these parameters, except" Level of consciousness" are assigned a score from 0 to 4 based on the actual values recorded from the patient corresponding to the range of values in the table below. For the level of consciousness: Aware/awake (0), Lethargy or irritability (1), Shallow coma (2), coma (3), brain death (4).

**Systemic Inflammatory Response scale (SIRS)**

| **Description** | **0 points** | **1 point** | **2 points** | **3 points** | **4 points** | **score** |
| --- | --- | --- | --- | --- | --- | --- |
| Heart beat | 60-100 | 55-59;  100-119 | 50-54;  120-140 | 41-49;  141-160 | <40;  >160 |  |
| MAP (mmHg) | 70-100 | 60-69;  101-110 | 50-59;  111-130 | 40-49;  131-159 | <40;  >160 |  |
| Respiratory rate/min | 12-20 | 9-12;  20-25 | 5-8;  26-35 | <5;  36-45 | 0;  >46 |  |
| SpO^2^（%） | >92 | 85-91 | 75-84 | 60-74 | <60 |  |
| Body temperature（℃） | 36.0-37.5 | 35-35.9;  37.5-38.5 | 34-34.9;  38.6-39.5 | 33-33.9;  39.6-40 | <33;  >40 |  |
| WBC(*10^9^/L) | 4.0-10.0 | 3.0-3.9;  10.1-14.9 | 2.0-2.9;  15-20.0 | 1.0-2.0;  20.1-30.0 | <1.0;  >30.1 |  |
| GLU (mmol/L) | 3.5-5.5 | 5.7-8.6 | 8.7-13.5 | 13.6-23 | >23 |  |
| Level of consciousness | Aware/awake | Lethargy or irritability | Shallow coma | coma | brain death |  |
| **Total score:** | | | | | |  |

**The 7-point ordinal scale**

**The 7-point ordinal scale** is consisting of seven separate categories. The categories range from 1-7,

1. not hospitalized with the resumption of normal activities;
2. patients are not hospitalized, but unable to resume normal activities;
3. patients are also non-ICU hospitalized patients, but they do not require supplemental oxygen;
4. patients are non-ICU hospitalized patients, requiring supplemental oxygen;
5. patients need high-flow oxygen but do not require ECMO and/or invasive mechanical ventilation;
6. patients need ICU hospitalization, require ECMO and invasive mechanical ventilation;
7. corresponds to death.
